# The Innovative Role of Nuclear Receptor Interaction Protein in Orchestrating Invadosome Formation for Myoblast Fusion

**DOI:** 10.1002/jcsm.13598

**Published:** 2024-09-25

**Authors:** Hsin‐Hsiung Chen, Chia‐Yang Lin, Ya‐Ju Han, Yun‐Hsin Huang, Yi‐Hsiang Liu, Wan‐En Hsu, Li‐Kai Tsai, Hsing‐Jung Lai, Yeou‐Ping Tsao, Hsiang‐Po Huang, Show‐Li Chen

**Affiliations:** ^1^ Graduate Institute of Microbiology, College of Medicine National Taiwan University Taipei Taiwan; ^2^ Department of Neurology National Taiwan University Hospital Taipei Taiwan; ^3^ Department of Ophthalmology Mackay Memorial Hospital Taipei Taiwan; ^4^ Graduate Institute of Medical Genomics and Proteomics, College of Medicine National Taiwan University Taipei Taiwan

**Keywords:** actin, invadosome, IQ domain, myoblast fusion, NRIP, WD40 domain

## Abstract

**Background:**

Nuclear receptor interaction protein (NRIP) is versatile and engages with various proteins to execute its diverse biological function. NRIP deficiency was reported to cause small myofibre size in adult muscle regeneration, indicating a crucial role of NRIP in myoblast fusion.

**Methods:**

The colocalization and interaction of NRIP with actin were investigated by immunofluorescence and immunoprecipitation assay, respectively. The participation of NRIP in myoblast fusion was demonstrated by cell fusion assay and time‐lapse microscopy. The NRIP mutants were generated for mechanism study in NRIP‐null C2C12 (termed KO19) cells and muscle‐specific NRIP knockout (NRIP cKO) mice. A GEO profile database was used to analyse NRIP expression in Duchenne muscular dystrophy (DMD) patients.

**Results:**

In this study, we found that NRIP directly and reciprocally interacted with actin both in vitro and in cells. Immunofluorescence microscopy showed that the endogenous NRIP colocalized with components of invadosome, such as actin, Tks5, and cortactin, at the tips of cells during C2C12 differentiation. The KO19 cells were generated and exhibited a significant deficit in myoblast fusion compared with wild‐type C2C12 cells (3.16% vs. 33.67%, *p* < 0.005). Overexpressed NRIP in KO19 cells could rescue myotube formation compared with control (3.37% vs. 1.00%, *p* < 0.01). We further confirmed that NRIP directly participated in cell fusion by using a cell–cell fusion assay. We investigated the mechanism of invadosome formation for myoblast fusion, which depends on NRIP–actin interaction, by analysing NRIP mutants in NRIP‐null cells. Loss of actin‐binding of NRIP reduced invadosome (enrichment ratio, 1.00 vs. 2.54, *p* < 0.01) and myotube formation (21.82% vs. 35.71%, *p* < 0.05) in KO19 cells and forced NRIP expression in KO19 cells and muscle‐specific NRIP knockout (NRIP cKO) mice increased myofibre size compared with controls (over 1500 μm^2^, 61.01% vs. 20.57%, *p* < 0.001). We also found that the NRIP mRNA level was decreased in DMD patients compared with healthy controls (18 072 vs. 28 289, *p* < 0.001, *N* = 10 for both groups).

**Conclusions:**

NRIP is a novel actin‐binding protein for invadosome formation to induce myoblast fusion.

## 1 Introduction

The nuclear receptor interaction protein (NRIP) (also named DCAF6 and IQWD1) consists of 860 amino acids, with seven WD40 repetitions and one IQ motif [1, 2]. NRIP interacts with various proteins to execute its multiple biological functions in different cells or tissues. For example, NRIP is an androgen receptor (AR)‐binding protein that mediates AR‐regulated gene expression [3]. NRIP can interact with Cullin 4 and Damage‐specific DNA binding protein 1 to prevent the ubiquitination and degradation of AR [4]. NRIP can also bind to calmodulin (CaM) through its IQ motif to activate downstream calcineurin (CaN) and CaM kinase II signalling [1], thereby regulating muscle contraction [5].

NRIP global knockout mice exhibit muscle dysfunction but remain vital [5]. Furthermore, muscle‐restricted NRIP‐knockout (cKO) mice show muscle dysfunction and retrograde motor neuron degeneration with an abnormal neuromuscular junction [6]. Additionally, the binding of NRIP to α‐actinin 2 (ACTN2) at the Z‐disc can facilitate F‐actin bundling. Correspondingly, NRIP deficiency disrupts sarcomere structural integrity, leading to cardiomyopathy [7]. After muscle injury, NRIP induction is essential for timely adult muscle regeneration; its deficiency delays repair capacity [5].

Myogenesis is the process of developing skeletal muscles during embryogenesis and post‐injury regeneration. Initially, myogenic progenitors are activated from quiescence to become myoblasts, followed by the proliferation and differentiation of myoblasts into myocytes and myotubes. As myotubes mature, they undergo secondary fusion to form myofibres bundled to take the shape of skeletal muscles [8, 9, 10, 11].

Myoblast fusion is fundamental in forming a nucleated syncytium to execute muscle function. Actin polymerization/depolymerization regulates this process between fusion‐competent myoblasts and founder cells during myogenesis. Muscle cells extend actin‐based lamellipodium and filopodium to recognize and contact neighbouring cells. Two fusion partners rearrange the actin cytoskeleton to form an F‐actin‐enriched and invasive podosome‐like structure (viz., invadosome) [12, 13]. Several actin‐binding proteins functioning in actin cytoskeleton remodelling are important for myoblast fusion, as they facilitate actin‐based protrusion, assist in actin polymerization, and guide the positioning of prefusion vesicle transport [14, 15, 16].

NRIP is induced after muscle injury, and NRIP‐KO mice exhibit more small‐sized myofibres post‐injury than wild‐type mice [5]. NRIP can directly interact with ACTN2, which is bundled with actin and required for myoblast fusion [7]. In this study, we investigated NRIP's role in myoblast fusion. Our results showed that NRIP is a novel actin‐binding protein, generating an invadosome‐like structure for myoblast fusion, which forms large‐sized myofibres.

## 2 Methods

### 2.1 Generation of NRIP‐Null C2C12 Myoblasts

The two guide RNAs for NRIP genome targeting were expressed by L‐Cas9n‐EGFP and R‐Cas9n‐puro, and the sequences of single‐guide RNAs (sgRNAs) were NRIP‐sgRNA‐F: 5′‐GCC CGC ACC UGU UGU GGG AC‐3′ and NRIP‐sgRNA‐R: 5′‐CUU GGG CUG GAG GAC CCG UCC‐3′. C2C12 cells were transfected with 2 μg plasmids (L‐Cas9n‐EGFP: R‐Cas9n‐puro = 3:1) by electroporation (1650 v/10 ms/3 pulses) with the Neon Transfection System Kit (Thermo Fisher Scientific). The cells were seeded overnight to 24‐well plates containing antibiotic‐free media. Cells were then selected with 3 μg/mL puromycin for 2 days and harvested. Some cells were analysed for editing efficiency, and others were single‐cell cultured in 96‐well plates.

### 2.2 Cell Culture and Transfection

C2C12, KO19, and 293T cells were cultured in DMEM supplemented with 10% FBS. For C2C12/KO19 differentiation, the culture medium was shifted to the differentiation medium, namely, DMEM supplemented with 2% horse serum. 293T cells and C2C12/KO19 cells were transfected with plasmids using jetPRIME (Polyplus) and the K2 transfection kit (Biontex), respectively, following the manufacturer's instructions. Cell proliferation was determined by the trypan blue exclusion assay. C2C12 or KO19 cells seeded in 10‐cm culture dishes (2 × 10^5^ cells per dish) with the growth medium were harvested at 1, 2, and 3 days and incubated with 0.4% trypan blue solution (Sigma). Cell numbers were counted using a haemocytometer under a microscope.

### 2.3 Immunofluorescence (IF) Staining

Cells were fixed with 2% paraformaldehyde and blocked with 2% bovine serum albumin for 30 min at room temperature. Samples were then incubated with the indicated primary antibodies [anti‐NRIP (GTX105954, GeneTex, 1:200), anti‐MyHC (ab124205, Abcam, 1:200), anti‐DsRed (632496, TaKaRa, 1:200), anti‐actin (Clone ID: NH3, ab205, Abcam, 1:200), anti‐Tks5 (sc‐376211, Santa Cruz, 1:100), anti‐cortactin (sc‐55579, Santa Cruz, 1:100), anti‐laminin (ab11575, Abcam, 1:1000), and anti‐EGFP (sc‐9996, Santa Cruz, 1:20)] at 4°C overnight. Then, the samples were incubated with fluorescent secondary antibodies (Cy3‐conjugated mouse anti‐rabbit, 488‐conjugated goat anti‐mouse, or 488‐conjugated goat anti‐rabbit, Jackson ImmunoResearch Laboratories) for 30 min and mounted in DAPI Fluoromount‐G (SouthernBiotech). The cell membrane was labelled with phalloidin (Sigma, 1:1000) for 1 h at room temperature. IF signals were recorded using a Zeiss Axioskop 40 Optical Microscope with an AxioCam 702 camera and Zeiss Zen Blue software.

### 2.4 Western Blot Analysis

For protein extraction, cells were pelleted by centrifugation in 600× *g* at 4°C for 30 min and lysed with RIPA buffer (20 mM Tris‐HCl, 150 mM NaCl, 1 mM EDTA, 1% NP‐40, pH 7.6). Crude extracts were centrifuged at 4°C to collect protein lysates from the supernatant. Protein lysates (50 μg) were subjected to western blotting with indicated primary antibodies: anti‐NRIP (A302‐434A, Novus, 1:2000), anti‐EGF receptor (#2646, Cell Signalling, 1:1000), anti‐DsRed (632496, TaKaRa, 1:10 000), anti‐actin (ab179467, Abcam, 1:10 000), anti‐EGFP (ab6556, Abcam, 1:10 000), anti‐flag (ab1162, Abcam, 1:10 000), anti‐MyHC (ab124205, Abcam, 1:2000), anti‐Tks5 (sc‐376211, Santa Cruz, 1:1000), anti‐cortactin (sc‐55579, Santa Cruz, 1:1000), anti‐His (66005‐1‐Ig, Proteintech, 1:10 000), anti‐GST (sc‐459, Santa Cruz, 1:10 000), anti‐PCNA (#2586, Cell Signalling, 1:10 000), and anti‐GAPDH (LF‐PA0212, AbFrontier, 1:10 000).

### 2.5 His and GST Pull‐Down Assays

For GST‐actin plasmid construction, the PCR‐amplified mCherry‐actin cDNA was subcloned into the pGEX‐4T‐1 vector (GE Healthcare). The His‐MBM‐NRIP plasmid was previously generated [1]. For His pull‐down assay, the His‐MBP and His‐MBP‐NRIP proteins were expressed in the *E. coli* Rosetta strain (Novagen) by isopropyl β–D‐1‐thiogalactopyranoside (1 mM) induction. The crude extracts (2 mg) were incubated with Ni‐NTA beads (Qiagen) at 4°C for 1 h. The eluted extracts were then incubated with GST‐actin and eluted with SDS sample buffer for subsequent western blotting. For the GST pull‐down assay, the GST and GST‐actin proteins (1 mg) generated from the *E. coli* BL21 strain (Novagen) were incubated with glutathione‐Sepharose 4B beads (Cytiva) at 4°C for 1 h, followed by elution and incubation with His‐MBP‐NRIP and eluted with SDS sample buffer for subsequent western blotting. In the NRIP mutant pull‐down assay for actin binding, bacteria‐produced His‐MBP‐NRIPs (NRIP‐FL, NRIP∆IQ, NRIP‐C, NRIP‐C‐∆WD6/7, and NRIP‐WD6/7) were incubated with F‐actin (AD99, cytoskeleton), followed by purification using Ni‐NTA beads and subjected to western blotting.

### 2.6 Immunoprecipitation Assay

The 293T cells were co‐transfected with pFlag‐NRIP and pmCherry‐actin, and then the protein lysates were subjected to immunoprecipitation assay. For mapping NRIP domains for actin binding, pmCherry‐actin was co‐transfected with pEGFP‐NRIP‐Full or its mutants (pEGFP‐NRIP‐C, pEGFP‐NRIP△IQ, pEGFP‐NRIP‐C‐△WD6/7, and Flag‐NRIP‐WD6/7) [1, 2] into 293T cells, and the immunoprecipitation assay was performed and followed by western blotting. For endogenous immunoprecipitation of NRIP and actin, protein lysates from 5‐day differentiated C2C12 myotubes were incubated with anti‐NRIP, anti‐actin, or the normal IgG control (rabbit IgG for anti‐NRIP; mouse IgG for anti‐actin). The immunoprecipitates were analysed by western blotting.

### 2.7 Nuclear, Cytosolic, and Membrane Protein Fractionation

Cytosolic and membrane proteins were extracted using the Mem‐Plus Membrane Protein Extraction Kit (Thermo 89842). Briefly, cells were pelleted by centrifugation at 300× *g* for 5 min, washed, lysed with a permeabilization buffer for 10 min at 4°C, and centrifuged for 15 min at 300× *g* to obtain the cytosolic fraction. Then, a solubilization buffer was added to cell pellets for 30 min with constant mixing and centrifugated at 16 000× *g* for 15 min to extract solubilized membrane‐associated proteins for subsequent western blotting. For nuclear extraction, the cell pellets were incubated with Benzonase nuclease for 15 min at room temperature. Subsequently, the nuclear extraction buffer containing 300 mM NaCl, 1.5 mM MgCl_2_, 5 mM HEPES pH 7.9, and 0.2 mM EDTA was added, and the mixture was further incubated for 10 min at 4°C. The samples were then centrifuged at 16 000× *g* for 10 min at 4°C to collect the nuclear extract.

### 2.8 Cell Fusion Assay

C2C12 cells were transfected with pmCherry, and KO19 cells were transfected with either pEGFP vector or pEGFP‐NRIP using the K2 transfection kit (Biontex) in growth medium for 24 h, followed by replacement with fresh growth medium and cultured for an additional 24 h. Then, the cells were trypsinized and mixed in a ratio of 1:1 (each cell line: 7.1 × 10^4^ cells/cm^2^) and seeded in 12‐well plates. The cells were shifted to a differentiation medium the next day and incubated for 12 days to form fused myotubes, followed by IF microscopy.

### 2.9 Time‐Lapse Microscopy

C2C12 myoblasts grown to 80% confluence were co‐transfected with pmCherry‐actin and pEGFP‐NRIP. We then trypsinized and replated the cells on the Matrigel‐coated cover glass to reach 50% confluence. For live cell imaging, cells in a chamber with phenol red‐free differentiation medium were observed at 37°C, 5% CO_2_ under a Laser TIRF/Spinning Disc Confocal Microscope (Zeiss). The cells with double‐positive signals (at least 10 cells for each experiment) were randomly selected to record the cell–cell fusion dynamic at 10‐min intervals for 6 h (Video S1).

### 2.10 AAV‐NRIP Generation and Injection

The PCR‐amplified DNA fragments of Flag‐tagged NRIP and its mutants (EGFP‐tagged NRIP‐C, NRIP‐C‐△WD6/7, and NRIP‐WD6/7) were subcloned into the AAV‐MCS vector. To generate AAV‐NRIP mutants or AAV‐EGFP, HEK293T cells were co‐transfected with the pAAV‐MCS‐NRIP or pAAV‐GFP, pAAV‐DJ/8, and the adenovirus helper plasmid pHelper using calcium phosphate transfection for 72 h. The AAV particles were purified by CsCl density‐gradient ultracentrifugation. AAV‐NRIP mutants or AAV‐EGFP (2 × 10^11^ vg each) were then injected into the bilateral hind limbs (gastrocnemius and tibialis anterior muscles) and forelimbs (triceps and biceps) of the 6‐week‐old NRIP cKO mice. Hence, each mouse received eight injections, totalling 1.6 × 10^12^ vg/mouse. The therapeutic effects were analysed at 16 weeks of age.

### 2.11 Animal Study

All animal procedures were reviewed and approved by the Institutional Animal Care and Usage Committee of the College of Medicine, National Taiwan University. The experimental mice were housed in the animal centre under a 12‐h light/dark cycle with free access to food and water.

### 2.12 Statistical Analysis

All statistical data were analysed using Prism (GraphPad Software). Data are presented as mean ± SD. Student's *t*‐test compared two groups, while one‐way ANOVA compared more than two data sets. *p* < 0.05 was considered statistically significant.

## 3 Results

### 3.1 NRIP Is a Novel Actin‐Binding Protein

**FIGURE 1:**
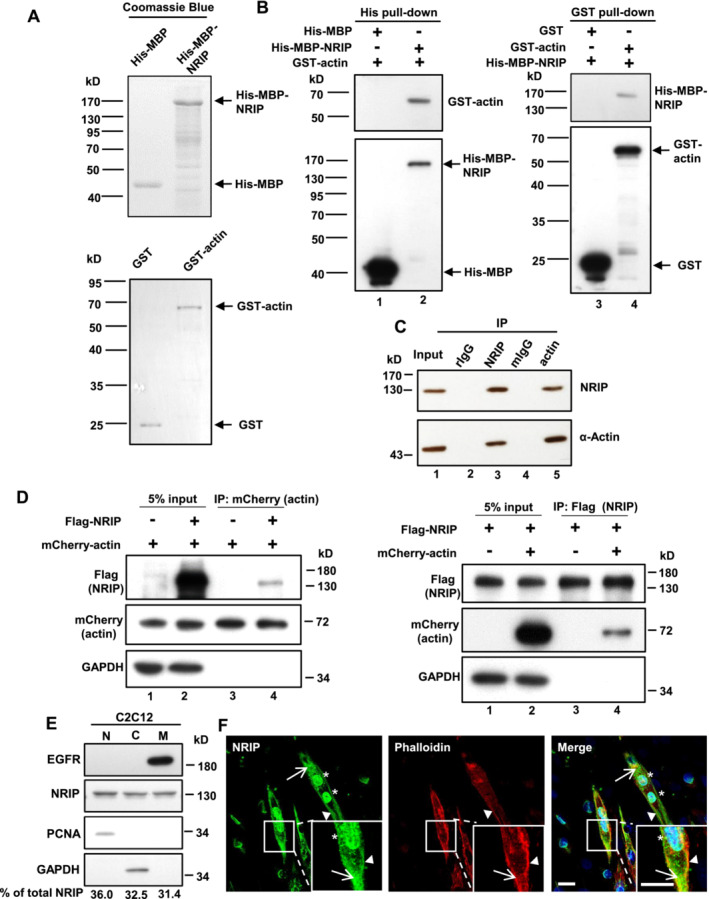
NRIP is an actin‐binding protein. (A) NRIP directly interacted with actin in vitro. Upper panel: His‐MBP and His‐MBP‐NRIP proteins were examined using Coomassie blue staining. The arrows indicate His‐MBP and His‐MBP‐NRIP. Lower panel: GST and GST‐actin proteins indicated by arrows. (B) Left panel: His pull‐down assy. Right panel: GST pull‐down assay. The pull‐downed extracts were analysed by western blotting with an anti‐His antibody to detect His‐MBP and His‐MBP‐NRIP and an anti‐GST antibody to detect GST‐actin. (C) NRIP interacted with actin in C2C12 myotubes. The protein extracts from C2C12 myotubes were immunoprecipitated with anti‐NRIP or anti‐alpha‐actin and immunoblotted with indicated antibodies. The rabbit normal IgG (rIgG) served as the antibody control for anti‐NRIP, while the mouse normal IgG (mIgG) was the control for anti‐alpha‐actin. (D) NRIP interacted with actin in cells. Cell extracts (1 mg) from 293T cells co‐transfected with Flag‐NRIP and mCherry‐actin were immunoprecipitated with anti‐mCherry (actin) (left panel) or with anti‐Flag (NRIP) (right panel) and immunoblotted with indicated antibodies. (E) Subcellular localization of NRIP in C2C12 cells. Nuclear (N), cytosolic (C), and membrane (M) fractions from C2C12 cells were extracted as described in Methods. EGFR was a positive control of membrane proteins, PCNA was a positive control for nuclear proteins, and GAPDH was a positive control for cytosolic proteins. % of total NRIP was determined as the intensity ratio of nuclear, cytosolic, or membrane NRIP to total NRIP (*N* = 3). (F) Endogenous NRIP was located at the plasma membrane, nucleus and cytoplasm. C2C12 myoblasts were differentiated for 4 days and stained with anti‐NRIP (green), phalloidin for the cell membrane (red, F‐actin staining), and DAPI for the nucleus (blue). Arrowheads: either NRIP or F‐actin at the plasma membrane. Arrows: NRIP in the cytoplasm. Asterisks: NRIP in the nucleus. Scale bar: 20 μm.

Considering NRIP's involvement in myogenesis and its multi‐interacting nature, we hypothesized that NRIP could affect myogenesis by directly interacting with actin. To verify this in vitro, a reciprocal pull‐down assay was performed using the plasmid expressing glutathione‐S‐transferase tagged actin (GST‐actin) and the plasmid expressing histidine‐maltose binding protein (His‐MBP) tagged NRIP (His‐MBP‐NRIP) [1]. The His‐MBP and His‐MBP‐NRIP proteins (Figure 1A, upper panel) were then incubated with GST or GST‐actin (Figure 1A, lower panel). The results showed that GST‐actin was pull‐downed with His‐MBP‐NRIP using nickel‐nitrilotriacetic acid (Ni‐NTA) beads (Figure 1B, left panel). Conversely, the His‐MBP‐NRIP was pull‐downed with GST‐actin using glutathione beads (Figure 1B, right panel). Hence, NRIP and actin exhibited reciprocal binding in vitro. To assess the interaction of NRIP and actin in mammalian cells, we performed endogenous immunoprecipitation in C2C12 myotubes. The protein extracts from five‐day differentiated C2C12 cells were immunoprecipitated with anti‐NRIP or anti‐alpha‐actin. Western blotting confirmed the interaction by detecting actin in the anti‐NRIP precipitate and, conversely, NRIP in the anti‐alpha‐actin precipitate (Figure 1C). Furthermore, we performed a co‐immunoprecipitation assay with Flag‐NRIP and mCherry‐actin plasmids co‐transfected into 293T cells. NRIP co‐precipitated with mCherry‐actin (Figure 1D, left panel) and vice versa (Figure 1D, right panel). Apart from cytosolic actin‐binding proteins, several membrane‐bound proteins are known to regulate myoblast fusion [17, 18, 19, 20, 21]. Thus, we examined the subcellular localization of NRIP in C2C12 cells by fractionating C2C12 myoblasts into nuclear, cytosolic, and membrane fractions. NRIP was localized in the nucleus, cytoplasm, and cell membrane at nearly equal proportions (36.0%, 32.5%, and 31.4%, respectively; Figure 1E). Additionally, immunofluorescence (IF) staining of C2C12 cells showed that NRIP colocalized with actin at the membrane (co‐stained with phalloidin, an F‐actin binding protein) (Figure 1F, arrowheads). NRIP was also localized at the nucleus and cytoplasm (Figure 1F, asterisks). Thus, NRIP directly interacted with actin in vitro and in cells, co‐localizing at the cell membrane.

### 3.2 NRIP Enhanced Myoblast Fusion

**FIGURE 2:**
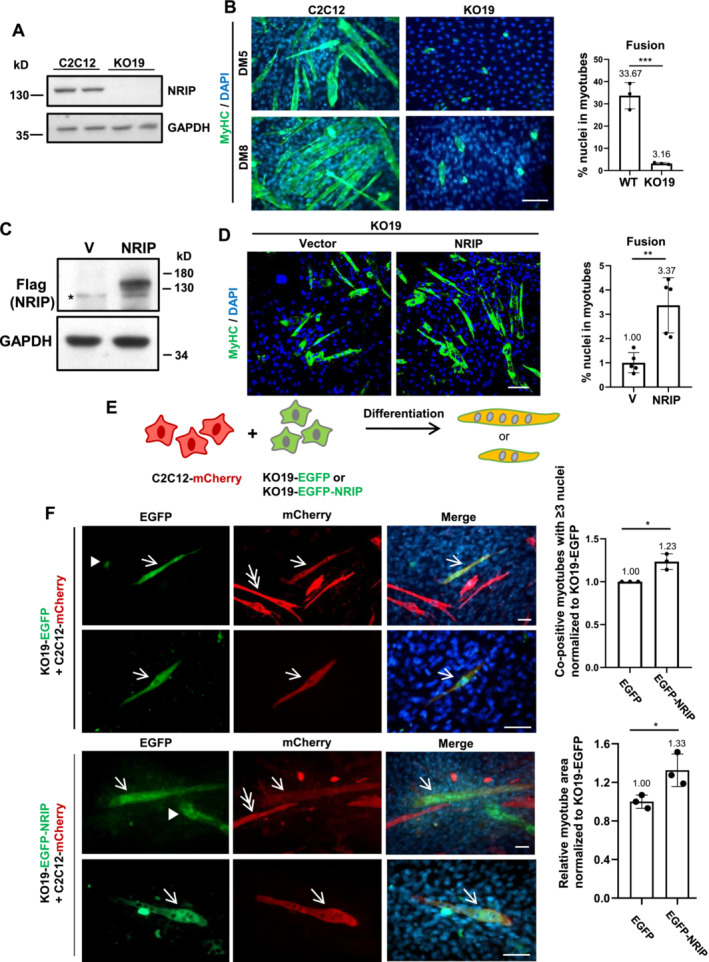
NRIP was involved in myoblast fusion. (A) Western blot analysis of NRIP in KO19 cells. (B) Left panel: KO19 cells (NRIP‐null C2C12 cells) undergoing myotube formation. C2C12 and KO19 cells were differentiated for 5 (DM5) or 8 days (DM8) and stained with anti‐MyHC (green) and DAPI for the nucleus (blue). Scale bar: 100 μm. Right panel: fusion index measured by the percentage of nuclei contained in MyHC^+^ myotubes (≥2 nuclei) to total nuclei per field (*N* = 3). (C) Western blot analysis showed the protein expression of Flag‐NRIP in KO19 cells. The asterisk (*) indicated the non‐specific band. (D) Left panel: NRIP overexpression in NRIP‐null cells rescued myotube formation. KO19 cells were transfected with Flag vector or Flag‐NRIP plasmid and stained with anti‐MyHC (green) and DAPI (blue) to assess myotube formation. Scale bar: 100 μm. Right panel: fusion index (*N* = 5). (E) A schematic illustration of cell fusion assay. (F) Left panel: IF staining showing the NRIP rescue increased myotube size and nuclei in cell–cell fusion assay. mCherry‐transfected C2C12 cells were co‐cultured with EGFP vector‐ or EGFP‐NRIP‐transfected KO19 cells for myotube differentiation and subsequently stained with anti‐EGFP (green) and anti‐DsRed for mCherry (red) to enhance the fluorescence intensity. Single green (arrowhead) or single red (double arrow) represented no fusion. Green and red co‐positive cells (arrow) indicated the fused myotubes. Scale bar: 100 μm. Upper right panel: quantitative analysis of cell–cell fusion. The ratio of co‐positive larger myotubes (≥3 nuclei) to the total co‐positive myotubes was calculated and normalized to that of the KO19‐EGFP group (*N* = 3). Lower right panel: quantitative analysis of myotube area. The myotube area was calculated using image J and normalized to that of the KO19‐EGFP group (*N* = 3). Data are mean ± SD. *p < 0.05, **p < 0.01, and ***p < 0.005; Student's *t*‐test.

To investigate the role of NRIP in myoblast fusion, we used the CRISPR‐Cas9 system (Figure S1) to generate NRIP‐null C2C12 cells (Figures 2A and S1). One clone of NRIP‐null C2C12 myoblasts (Figure S1C), KO19, was chosen for the following study. We found no significant difference in the proliferation rates of KO19 and wild‐type C2C12 cells before comparing the number of MyHC^+^ myotubes (Figure S2A). IF microscopy showed much fewer MyHC^+^ cells in KO19 compared with C2C12 cells at both 5‐ and 8‐days post‐differentiation (Figure 2B, left panel). KO19 cells barely formed myotubes 5 days post‐differentiation and produced only few myotubes 8 days post‐differentiation. We measured the fusion index, defined as the percentage of nuclei contained within MyHC^+^ myotubes (≥2 nuclei) relative to the total number of nuclei per field in myoblasts on day 8. KO19 cells showed a reduced fusion index compared with C2C12 cells (3.16% vs. 33.67%, *p* < 0.005, Figure 2B, right panel). To examine whether NRIP could restore myoblast fusion in NRIP‐null cells, KO19 cells were transfected with a plasmid expressing Flag‐NRIP, with the expression confirmed by IF staining (Figure S2B) and western blotting (Figure 2C). IF microscopy revealed that KO19/NRIP cells had larger, longer, and more myotubes than KO19/vector cells (Figure 2D, left panel) and exhibited enhanced myoblast fusion compared with KO19/vector cells (3.37% vs. 1.00%, *p* < 0.01, Figure 2D, right panel), suggesting that re‐expression of NRIP in KO19 cells restored myotube formation. To further demonstrate NRIP's participation in cell–cell fusion, we transfected C2C12 cells with a mCherry vector and KO19 cells with either an EGFP vector or EGFP‐NRIP. C2C12 and KO19 cells were then co‐cultured for differentiation into myotubes (Figure 2E). EGFP^+^ only or mCherry^+^ only cells indicated non‐fusion, while co‐positive cells indicated fusion. The fused KO19/EGFP‐NRIP and C2C12/mCherry cells formed larger myotubes with more nuclei (Figure 2F, left panel, arrows) compared with the control (KO19/EGFP‐C2C12/mCherry fusion cells), as confirmed by quantification: the normalized ratio of co‐positive larger myotubes (≥3 nuclei) to total co‐positive myotubes was higher in the EGFP‐NRIP group compared with the EGFP group (1.23: 1, *p* < 0.05; Figure 2F, upper right panel). Furthermore, the myotube area of fused KO19/EGFP‐NRIP and C2C12/mCherry cells was larger than that of the control (KO19/EGFP and C2C12/mCherry) fusion cells (1.33:1, *p* < 0.05; Figure 2F, lower right panel). Thus, NRIP enhanced cell–cell fusion to form larger myotubes.

### 3.3 NRIP Facilitated Invadosome Formation for Myoblast Fusion

**FIGURE 3:**
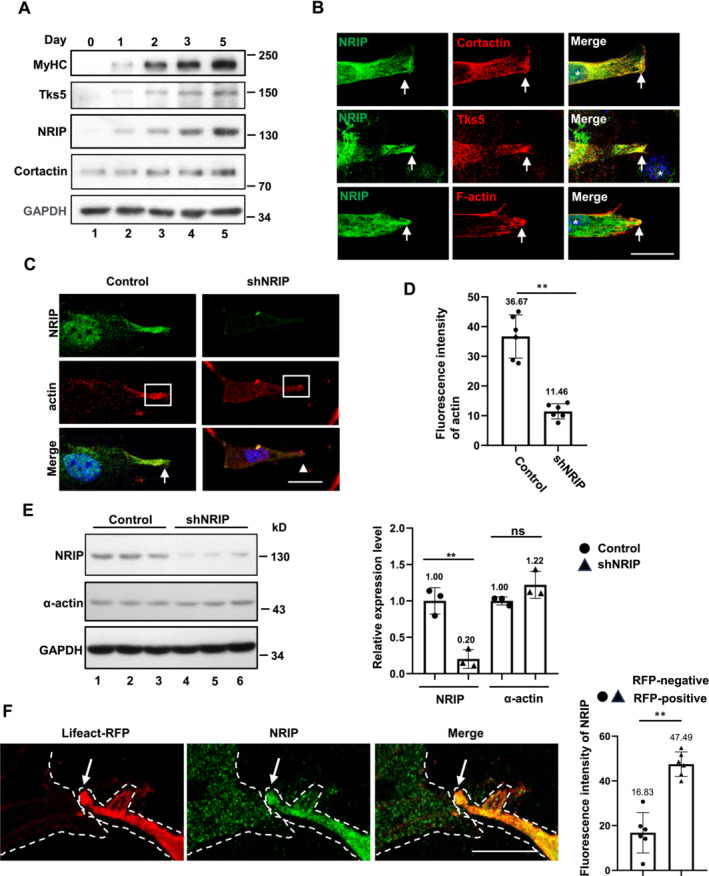
Co‐enrichment of NRIP and invadosome components to form invadosome for myoblasts fusion. (A) The temporal expression profiles of NRIP and invadosome proteins (Tks5 and cortactin) during C2C12 differentiation. MyHC as a differentiation marker. The GAPDH was a loading control. (B) Endogenous NRIP and other invadosome component proteins were situated at the tips during myoblast fusion. The enriched NRIP colocalized with cortactin (upper), Tks5 (middle), and actin (lower). Green: NRIP; red: each invadosome component; blue: DAPI for nuclear staining. The arrows: cell tips. Scale bar: 100 μm. (C) Knockdown of *NRIP* reduced the tip distribution of actin. The C2C12 cells transfected with the pSuper‐shNRIP plasmid were stained with anti‐actin (red) and anti‐NRIP (green). Scale bar: 100 μm. Arrow: the location of enriched NRIP and actin. Arrowhead: the area of dispersed actin. Scale bar: 100 μm. Box: C2C12 cell tips. (D) Average fluorescence intensity of actin at the tips of NRIP‐knockdown C2C12 cells and control cells. The area of the tip is about 480 μm^2^ in C2C12 cells with or without NRIP knockdown (*N* = 6). (E) The alpha‐actin protein expression was not affected by NRIP knockdown. Left panel: western blot analysis of NRIP and alpha‐actin in C2C12 cells with or without NRIP knockdown at differentiated day 5. GAPDH: loading control. Right panel: Quantification of NRIP and alpha‐actin proteins in the left panel (*N* = 3). (F) Asymmetric colocalization of NRIP and actin during myoblast fusion. Left panels: The C2C12 cells were transfected with Lifeact‐RFP (for F‐actin labeling) on day 3 of differentiation and stained for NRIP. The dashed lines represent the cell periphery of two cells (the left as the receiving cell; the right as the attacking cell containing invadosome). Right panel: quantification of the average NRIP fluorescence intensity in the tips of Lifeact‐RFP‐positive and Lifeact‐RFP‐negative cells (*N* = 6). The average area of the tip was about 480 μm^2^ in both Lifeact‐RFP‐positive and Lifeact‐RFP‐negative cells. Scale bar: 100 μm. Data are mean ± SD. **p < 0.01; ns, no significance; Student's *t*‐test.

Invadosomes, actin‐enriched protrusions crucial for invasion and fusion pore formation, can be formed in C2C12 cells by the collaboration of actin, Tks5 (invadosome scaffold protein), and cortactin (actin‐binding protein, also an invadosome marker) [22, 23, 24, 25]. Hence, we hypothesized that NRIP might be involved in invadosome formation through actin binding. We examined NRIP, Tks5, and cortactin expression patterns during C2C12 differentiation. The NRIP levels aligned with the gradually increasing expression patterns of MyHC, Tks5, and cortactin proteins (Figure 3A) during myoblast differentiation [23]. IF microscopy showed that NRIP was colocalized with cortactin, Tks5, and F‐actin at the tips of C2C12 cells (Figure 3B; arrows). To confirm whether NRIP is required for invadosome formation, we transfected pSuper‐shNRIP into C2C12 cells to knock down the endogenous NRIP expression, as shown by IF staining (Figure 3C) and western blot analysis (control vs. shNRIP = 1.00: 0.20; Figure 3E). We observed a significant decrease in the average fluorescence intensity of both actin (11.46 vs. 36.67, *p* < 0.01; Figure 3C,D) and Tks5 (15.75 vs. 32.17, *p* < 0.05; Figure S3A, B) at the tips of NRIP‐knockdown C2C12 myoblasts compared with controls, while total alpha‐actin and Tks5 protein levels remained unaffected (Figures 3E and S3C). The total protein levels of Tks5 and cortactin in KO19 cells were comparable to those in C2C12 cells (Figure S3D). Moreover, restoring NRIP expression in KO19 cells did not affect the total protein levels of Tks5 and cortactin (Figure S3D). To further investigate the effect of complete NRIP deprivation on the tip localization of invadosome proteins, actin and Tks5 signals at KO19 cell tips were compared with those of wild‐type C2C12 cells, revealing a significant reduction in both actin (13.03 vs. 45.77; Figure S3E, F) and Tks5 (12.50 vs. 42.60; Figure S3G, H) in KO19 cells. Furthermore, we used a Lifeact‐RFP labeling assay to distinguish the localization pattern of NRIP in attacking and receiving cells during myoblast fusion. The 17‐amino‐acid Lifeact peptide, derived from the actin‐binding protein 140 of *S. cerevisiae* [26], specifically binds to F‐actin, which is highly expressed in attacking cells during myoblast fusion [23]. IF microscopy showed that NRIP was enriched in attacking cells expressing Lifeact‐RFP and was dispersed in receiving cells among closely positioned, 3‐day differentiated myoblasts (Figure 3F, left three panels). The average endogenous NRIP signal was higher at the tips of Lifeact‐RFP‐positive cells than those of Lifeact‐RFP‐negative cells (47.49 vs. 16.83, *p* < 0.01; Figure 3F, right panel). Together, NRIP was a novel component of the invadosome and crucial for invadosome formation through actin‐binding.

### 3.4 NRIP Participated in Invadosome Protrusion During Myoblast Fusion

**FIGURE 4:**
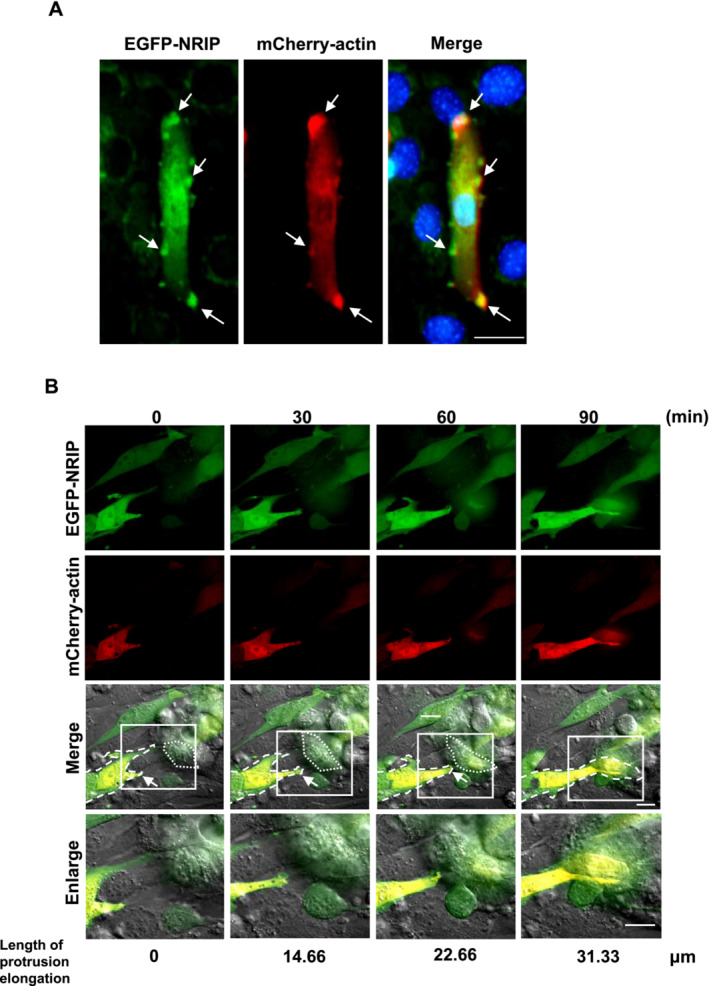
The dynamics of NRIP and actin expression in invadosome formation during myoblast fusion. (A) Finger‐like structures enriched for EGFP‐NRIP and mCherry‐actin in differentiated C2C12 cells. IF showed EGFP‐NRIP and mCherry‐actin were colocalized (indicated by arrows) at multiple invadosomes of C2C12 myotubes. DAPI (blue) for nuclei. Scale bar: 100 μm. (B) The dynamics of NRIP localization along with the invadosome protrusion during myoblast fusion. White arrows of merged images indicated the expression of EGFP‐NRIP along with mCherry‐actin in the invadosome structure of attacking cells. Boxed regions were magnified and shown in the lowest panel (Enlarge). Dash lines: attacking cells. Dot lines: receiving cells. Below the images, the lengths of protrusion elongations from the attacking cell during myoblast fusion are displayed. Scale bar: 20 μm.

To further demonstrate whether NRIP participated in invadosome protrusion, we used time‐lapse microscopy to analyse the dynamics of NRIP localization in C2C12 myoblasts co‐transfected with EGFP‐NRIP and mCherry‐actin during myoblast fusion. IF staining showed that EGFP‐NRIP was colocalized with mCherry‐actin to form the invadosome (Figure 4A, arrows). Sequentially, EGFP‐NRIP and mCherry‐actin concentrated to form foci in the C2C12 cell cytoplasm (0 min), which then protruded towards the cell membrane, creating a protrusive invadosome (30 min). The invadosome elongated and approached the receiving cell (60 min) before making contact, leading to the diffusion of EGFP‐NRIP and mCherry‐actin into the receiving cell's cytoplasm (90 min) (Figure 4B and Video S1). Furthermore, we observed a gradual increase in the length of protrusion elongation from the attacking cell during myoblast fusion: 14.66 μm at 30 min, 22.66 μm at 60 min, and 31.33 μm upon contact with the receiving cell (Figure 4B).

### 3.5 NRIP Domains for Actin Binding Were Also Essential for Invadosome Formation

**FIGURE 5:**
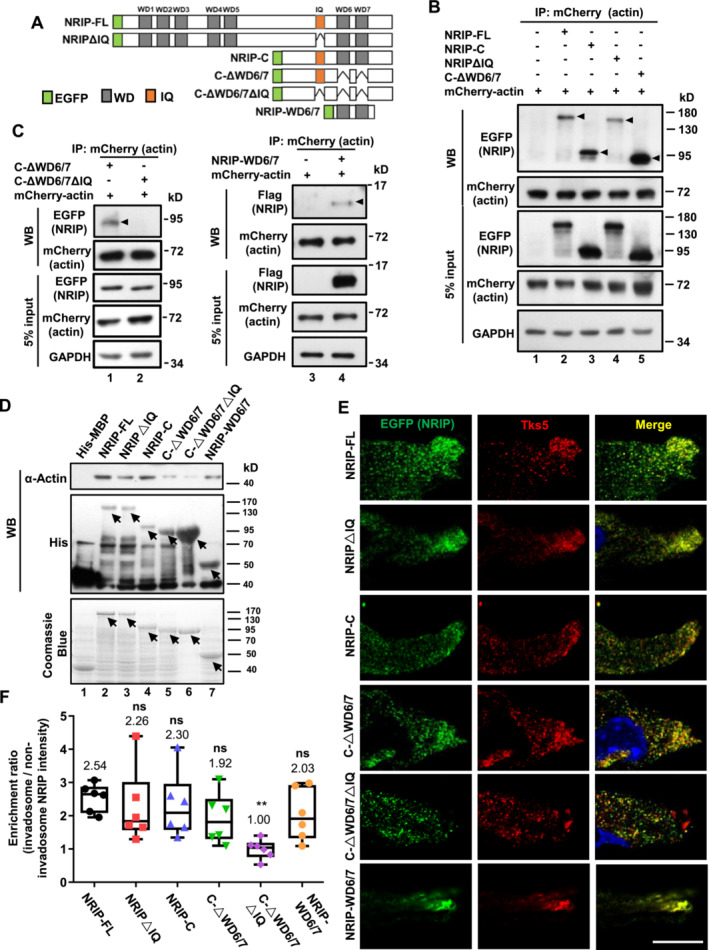
The correlation of NRIP‐actin binding with invadosome formation using NRIP mutant constructs. (A) Schematic illustration of EGFP‐tagged NRIP and various NRIP deletion constructs: NRIP‐FL, NRIPΔIQ, NRIP‐C, C‐ΔWD6/7, C‐ΔWD6/7ΔIQ, and NRIP‐WD6/7. (B) Domain mapping of the NRIP‐actin interaction. 293T cells were co‐transfected with individual NRIP truncated mutants and mCherry‐actin plasmid for immunoprecipitation assay with an anti‐mCherry antibody. Arrowheads indicate the precipitated NRIP by actin. GAPDH as a loading control. (C) Either WD6/7 domains or the IQ domain of NRIP could interact with actin in cells. C‐ΔWD6/7 (lane 1) and NRIP‐WD6/7 (lane 4), but not C‐ΔWD6/7ΔIQ (lane 2), were precipitated with actin. (D) NRIP‐WD6/7 domains were responsible for direct actin binding, as shown by the His pull‐down assay. Lower panel: Coomassie blue staining. The arrows indicate His‐MBP and His‐MBP‐NRIP mutants' proteins. Upper two panels: immunoblotting with an anti‐alpha‐actin or anti‐His antibody. (E) NRIP were localized at invadosomes through actin interaction. NRIP‐null KO19 cells transfected with EFGP‐NRIP mutants were stained with anti‐EGFP (NRIP, green) and anti‐Tks5 (invadosome marker, red) antibodies at differentiation day 3 for IF analysis. DPAI (blue) for nuclear stain. Scale bars, 20 μm. (F) Quantification of the relative abundance of NRIP mutants within the invadosome based on the data in panel (E). The enrichment ratios of NRIP mutants in the invadosome were quantified by dividing the NRIP intensity within Tks5‐positive foci by the NRIP intensity in the non‐invadosome area (*N* = 6). Data are mean ± SD. *p < 0.05; one‐way ANOVA.

We then determined the domains of NRIP essential for actin binding and explored the correlation between actin binding and invadosome formation through NRIP mutant analysis. We generated a series of EGFP‐tagged NRIP and NRIP deletion mutants: NRIP‐FL (full length), NRIP‐N (N fragment containing the first five WD40 domains without the IQ motif), NRIP‐C (C fragment containing two WD40 domains and the IQ motif), NRIPΔIQ (full length without the IQ motif), C‐ΔWD6/7 (C fragment without WD40 domains but with the IQ motif), C‐ΔWD6/7ΔIQ (C fragment without WD40 domains and the IQ motif), and NRIP‐WD6/7 (the sixth and seventh WD40 domains in NRIP‐C fragment) (Figures 5A and S4A). Each NRIP mutant was co‐transfected with mCherry‐actin into 293T cells and subjected to a co‐immunoprecipitation assay with an anti‐mCherry antibody. The results revealed that NRIP‐FL, NRIP‐C, NRIPΔIQ, and C‐ΔWD6/7 could bind to actin (Figure 5B). The NRIP‐N fragment (Figure S4A) could also bind to actin (Figure S4B), but in this study, we focused on analysing cytosol‐located NRIP mutants (e.g., NRIP‐C, NRIPΔIQ, and C‐ΔWD6/7), as the NRIP‐N fragment is located in the nucleus of 293T cells [1]. To further clarify the need for the WD40 domain and IQ motif for actin binding, we co‐transfected the C‐ΔWD6/7ΔIQ, C‐ΔWD6/7, or NRIP‐WD6/7 with mCherry‐actin in 293T cells and performed co‐immunoprecipitation, which showed loss of actin‐binding ability in C‐ΔWD6/7ΔIQ compared with C‐ΔWD6/7 (Figure 5C, left panel) and NRIP‐WD6/7 (Figure 5C, right panel). To further confirm the direct binding between the NRIP WD40 domain and actin, an in vitro pull‐down assay was performed. *E. coli* ‐generated His‐tagged NRIP mutant proteins (Figure 5D, lower panel) were incubated with bovine cardiac alpha‐actin and pulled down by nickel‐charged affinity resin for western blotting. The results showed that actin could be pulled down by NRIP containing WD40 (NRIP‐FL, NRIP‐C, NRIPΔIQ, and NRIP‐WD6/7) (Figure 5D). In contrast, NRIP without the WD6/7 domain (C‐ΔWD6/7 and C‐∆WD6/7∆IQ) lost most actin‐binding capacity (Figure 5D, lanes 5 and 6). As the IQ domain has been reported to bind to ACTN2 [7], the results suggested that NRIP relied predominantly on its WD40 6/7 domains for direct interaction with actin. To further investigate the correlation of NRIP's actin‐binding ability and invadosome formation and avoid endogenous NRIP's interference, we examined the expression and distribution of NRIP mutants and Tks5 in differentiated NRIP‐deficient KO19 cells (Figure 5E). The IF intensity of C‐ΔWD6/7ΔIQ in Tks5‐enriched invadosomes was significantly decreased compared with NRIP‐FL (1.00 vs. 2.54, *p* < 0.01, Figure 5E,F). The other mutants, such as NRIP‐C, NRIPΔIQ, C‐ΔWD6/7, and NRIP‐WD6/7, which possess the actin‐binding ability, showed no significant differences in their expressions and distributions in invadosomes compared with NRIP‐FL (Figure 5E,F). Similarly, the distribution of C‐ΔWD6/7ΔIQ in invadosome was decreased compared with NRIP‐FL in C2C12 myotubes (0.91 vs. 2.59, *p* < 0.05, Figure S5A, B). In summary, NRIP is required for invadosome formation by either directly interacting with actin through WD40 domains or indirectly through the IQ domain.

### 3.6 Correlation Between NRIP's Actin‐Binding Ability and Myotube Formation Rate

**FIGURE 6:**
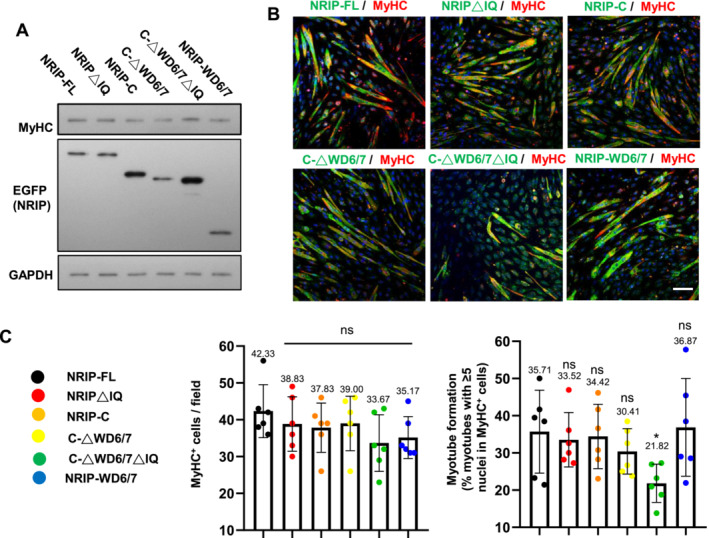
The NRIP domain with actin‐binding ability facilitated myotube formation. (A) The expression of MyHC and NRIP mutants in KO19 cells transfected with NRIP mutants. KO19 cells were transfected with various NRIP mutants, followed by western blotting with anti‐EGFP (NRIP mutants) and anti‐MyHC antibodies. GAPDH as a loading control. (B) Myotube formation in myoblasts expressing various NRIP mutants. KO19 cells were transfected with NRIP mutants and then subjected to IF stain with anti‐EGFP (NRIP, green) and anti‐MyHC (red) antibodies. DAPI for nuclear stain (blue). Scale bar: 100 μm. (C) Quantitation of MyHC^+^ cells and myotube formation in NRIP mutants in panel (B). Left panel: the numbers of MyHC^+^ cells per field for different NRIP mutants. Right panel: the percentages of myotubes (≥5 nuclei in MyHC^+^ cell) to total MyHC^+^ cells for different NRIP mutants. The data in each mutant represent six independent experiments (three random fields per experiment). Data are mean ± SD. *p < 0.05; ns, no significance; one‐way ANOVA.

To further investigate the correlation between NRIP‐actin interaction and the formation of multinucleated myotubes, we assessed MyHC expression in KO19 cells transfected with NRIP and mutants. Western blotting showed comparable MyHC protein levels across mutants (Figure 6A). IF analysis for MyHC indicated no significant difference in the number of MyHC^+^ cells among NRIP mutants (Figure 6B,C, left panel). Furthermore, the myotube formation rate was determined by calculating the proportion of MyHC^+^ myotubes with ≥5 nuclei among total MyHC^+^ cells for each mutant. Loss of actin‐binding ability in NRIP (C‐ΔWD6/7ΔIQ mutant) significantly decreased myotube formation compared with NRIP‐FL, while other mutants with actin‐binding abilities showed similar myotube formation rates as NRIP‐FL (Figure 6C, right panel). Similarly, C2C12 cells transfected with C‐ΔWD6/7ΔIQ showed a decrease in myotube formation compared with NRIP‐FL, with MyHC expression unaffected (Figure S6). Thus, forced NRIP expression in NRIP‐null cells rescued myotube formation, while loss of actin‐binding in NRIP reduced this ability, suggesting the requirement of NRIP‐actin interaction for myotube formation. To determine whether the loss of actin‐binding ability and reduced myotube formation in NRIP mutants were due to changes in the cellular localization of NRIP mutants, KO19 cells were transfected with NRIP mutants and stained with an anti‐EGFP antibody for cellular localization analysis (Figure S7A). The ratio of fluorescence intensity in the cytoplasm and nucleus for each NRIP mutant, compared with NRIP‐FL, was quantified and showed no significant difference (Figure S7B). Hence, the loss of actin‐binding ability and reduced myotube formation in the NRIP (C‐ΔWD6/7ΔIQ mutant) were not due to changes in cellular localization.

### 3.7 Rescue of NRIP Expression Via Adeno‐Associated Virus (AAV)‐NRIP Increased Myofibre Size in Muscle‐Specific NRIP Knockout (NRIP cKO) mice

**FIGURE 7:**
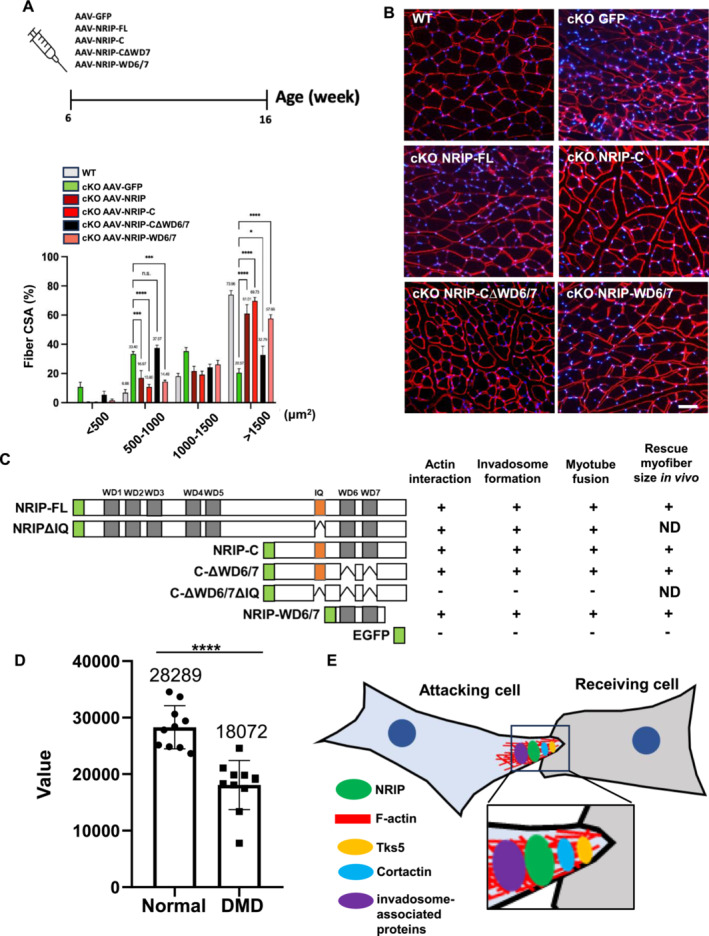
The AAV‐NRIP mutants with actin‐binding ability increased the myofibre size of NRIP cKO mice. (A) Schematic protocol for gene therapy in NRIP cKO mice via AAV‐NRIP mutants. (B) Right: the myofibre size of gastrocnemius muscles from NRIP cKO mice treated with various AAV‐NRIP mutants at the age of 16 weeks. The gastrocnemius muscles were stained with an anti‐laminin antibody to delineate the boundaries of myofibres. DAPI is for nuclear counterstain. Scale bar: 50 μm. Left: quantification of myofibre size. *N* = 3 for each group. Data are mean ± SEM. *p < 0.05, **p < 0.01, ***p < 0.001 and ns, not significant, analysed by Student's *t*‐test. (C) Summary of the relationship between NRIP mutants and their effects on actin‐binding, invadosome formation, myotube fusion, and myofibre size based on the results in Figures 5, 6, 7. ND, not determined. (D) *NRIP* expression in the muscles of DMD patients and normal controls using a GEO profile (accession number: GDS610). The RNA of quadriceps biopsies from DMD patients (*N* = 10) and unaffected controls (*N* = 10) were extracted and hybridized to HG‐U95 GeneChip. The value was measured from Affymetrix/Hewlett‐Packard G2500A Gene Array Scanner [27]. Data are mean ± SEM. ****p < 0.001 by Student's *t*‐test. (E) Schematic illustration depicting NRIP interaction with F‐actin at invadosome for myoblast fusion.

To investigate the role of NRIP's actin‐binding ability in myotube formation in vivo, we characterized myofibre size in 6‐week‐old NRIP cKO mice receiving intramuscular injection with different AAV‐delivered NRIP mutants (Figure 7A). The Flag‐tagged NRIP‐FL and EGFP‐tagged NRIP mutants were successfully expressed in the skeletal muscles of NRIP cKO mice (Figure S8). The myofibre cross‐section areas (CSA) were classified as under 500 μm^2^, 500–1000 μm^2^, 1000–1500 μm^2^, and over 1500 μm^2^. In the CSA range between 500 and 1000 μm^2^, mice treated with AAV‐NRIP‐FL, AAV‐NRIP‐C, or AAV‐NRIP‐WD6/7 showed a lower percentage compared with those treated with AAV‐GFP, while AAV‐NRIP‐C‐∆WD6/7 group showed no significant difference (Figure 7B). In contrast, mice treated with all NRIP mutants showed a significantly higher percentage of myofibres over 1500 μm^2^ compared with controls (Figure 7B). The results suggested that NRIP had a rescuing effect on the myofibre size of NRIP cKO mice via its C‐terminal WD40 domain. Furthermore, NRIP cKO mice exhibited a higher percentage of myofibres with central nuclei compared with wild‐type mice (3.58% vs. 1.41%, *p* < 0.01, Figure S9). Treatment with AAV‐NRIP‐FL, AAV‐NRIP‐C, or AAV‐NRIP‐WD6/7, but not AAV‐NRIP‐C‐∆WD6/7, resulted in a reduction of myofibres with central nuclei compared with AAV‐GFP (Figure S9). Therefore, the NRIP C‐terminal WD40 domain also had a rescuing effect on myofibre damage in NRIP cKO mice. Collectively, NRIP interacts with actin through the WD40 domain and IQ motif, enhancing invadosome formation, myoblast fusion, and ultimately myofibre size both in vitro and in vivo (Figure 7C). A reduction in myofibre size is the prominent phenotype of neuromuscular diseases such as Duchenne muscular dystrophy (DMD) and limb‐girdle muscular dystrophy (LGMD). Considering the downregulation of NRIP in LGMD muscles, we were prompted to analyse the NRIP expression in DMD patients. The data (accession number: GDS610) from the Gene Expression Omnibus (GEO) database [27] showed that *NRIP* expression was significantly decreased in the DMD muscles compared with healthy muscles (Figure 7D), suggesting that the reduced myofibre size in DMD might be associated with reduced NRIP expression. Finally, we presented a projected model illustrating the role of NRIP in myoblast fusion (Figure 7E).

## 4 Discussion

In this study, we demonstrated that NRIP directly interacted with actin in vitro and in C2C12 cells. NRIP‐null C2C12 cells exhibited a decreased fusion index, which could be rescued by re‐expression of NRIP. This aligns with our previous report [5], indicating excessive small myofibres during muscle regeneration in NRIP‐deficient mice. We also showed that NRIP was colocalized with other components of invadosome and was required for invadosome protrusion. Notably, we identified NRIP domains responsible for actin binding and assessed their correlation with invadosome and myotube formations in C2C12 cells, as well as myofibre size in NRIP cKO mice.

NRIP consists of 860 amino acids, with seven WD40 repeats and one IQ motif [1, 2]. WD40 domains are frequently involved in protein–protein interactions. They form seven‐blade‐shaped structures and exhibit β‐propeller architectures [28]. WD40 domain‐containing proteins can bind to multiple proteins, thereby regulating various cellular functions, such as cytoskeletal assembly and transcriptional regulation [28]. Several WD40‐containing proteins are actin‐binding proteins. For example, coronin, with five WD40 domains, enhances endocytosis and cell motility through interacting with actin [29], while actin‐interacting protein 1, containing two WD40 β‐propellers, promotes the disassembly of actin filaments [30]. Here, NRIP, containing seven WD40 domains [2], interacted with actin through its N‐terminal and C‐terminal WD40 domains. We further found that the C‐terminal WD40 domain (NRIP‐WD6/7) was sufficient to interact with actin. On the other hand, through its IQ domain, NRIP can bind to ACTN2 [7], which in turn interacts with actin for actin‐bundling formation [31]. Hence, we speculated that NRIP directly interacted with actin via the WD40 domain or indirectly via the IQ domain to enhance invadosome formation for myoblast fusion.

NRIP is a multifunctional protein whose function depends on its subcellular location. We demonstrated that NRIP was a membrane protein by IF analysis, with biochemical fractioning revealing approximately 50% in the membrane fraction. This supports our previous finding about NRIP's colocalization with acetylcholine receptors at the neuron muscular junction [6]. NRIP is also present in the cytosol, which is consistent with our previous report of NRIP's interaction with CaM or ACTN2 at the cytosolic sarcomere Z‐disc to activate muscle‐promoting calcineurin (CaN) and CaM kinase II signalling [32] and to stabilize the Z‐disc structure [5, 7], respectively. Moreover, we have detected NRIP expression in the nucleus, where it interacts with AR and serves as a transcriptional cofactor to augment AR target gene expression [2, 3]. Here, our results indicated that NRIP promoted invadosome formation and myoblast fusion via actin‐binding without affecting the expression of invadosome proteins (Tks5 and actin) and MyHC. However, we cannot rule out the possibility of NRIP as a transcriptional cofactor in regulating myogenesis, which may warrant further investigation. Similarly, β‐catenin plays a dual role as a transcriptional coactivator in the nucleus, forming a complex with TCF/LEF‐1 for developmental gene expression and maintaining cytoskeleton stability by anchoring cadherins to actin filaments in the cadherin–catenin complex [33, 34]. Future studies should explore the subcellular location of NRIP in various tissues and elucidate its novel functions there. Additionally, further investigations are needed to determine whether NRIP regulates MyoD/myogenin through nuclear transcriptional activation or cytosolic CaN‐CaM signalling.

It has been reported that DMD and LGMD patients have smaller myofibre sizes than normal controls [35, 36, 37]. Moreover, the myoblasts isolated from DMD patients displayed a reduced fusion index compared with healthy myoblasts [38]. Considering NRIP's involvement in myoblast fusion/myotube formation, we analysed the GEO data and found a significant downregulation of *NRIP* in DMD patients. This warrants further investigation into whether NRIP could be a potential therapeutic target for DMD in the future. Interestingly, the presence of centrally located nuclei in NRIP‐deleted muscle fibres suggests that the deletion of NRIP leads to muscle fibre fragility and damage, thereby causing an increased need for muscle regeneration, similar to that seen in several muscle disorders [39], including DMD [40].

In sum, NRIP plays a crucial role in myoblast fusion. This novel actin‐binding protein is actively involved in the formation of a protrusive invadosome, leading to an increased proportion of fused myotubes with multiple nuclei. NRIP interacted with actin in two ways: direct interaction via its WD40 domains and indirect interaction through ACTN2 via its IQ domain. Furthermore, we demonstrated that NRIP could increase myofibre size via its WD40 domains in vivo and in cells. Hence, NRIP holds physiological significance in increasing myofibre size and suggests its potential as a therapeutic target for DMD, wherein decreased *NRIP* expression has been observed. Thus, NRIP is a novel actin‐binding protein that orchestrates invadosome formation, facilitates myotube fusion, and ultimately enhances the generation of large‐sized myotubes.

## Conflicts of Interest

The authors declare no conflicts of interest.

## Supporting information


**Data S1.** Supporting Information

**Video S1.** Time‐lapse microscopy for NRIP and actin during invadosome structure formation (Figure 4B).
