# Supplementary material for: The Innovative Role of Nuclear Receptor Interaction Protein in Orchestrating Invadosome Formation for Myoblast Fusion
Source: J Cachexia Sarcopenia Muscle. 2024 Sep 25;15(6):2559–73. doi: 10.1002/jcsm.13598 (PMC11634477; doi:10.1002/jcsm.13598)
Supplement: Supplementary file 1 — Data S1. Supporting Information [file JCSM-15-2559-s001.pdf]

Supplementary Figure Legends

Journal of Cachexia, Sarcopenia and Muscle

The Innovative Role of Nuclear Receptor Interaction Protein in Orchestrating Invadosome Formation for Myoblast Fusion.

Hsin-Hsiung Chen[1], Chia-Yang Lin[1], Ya-Ju Han[1], Yun-Hsin Huang[1], Yi-Hsiang Liu[1], Wan-En Hsu[1], Li-Kai Tsai[2], Hsing-Jung Lai[2], Yeou-Ping Tsao[3], Hsiang-Po Huang[4,*] & Show-Li Chen[1,*]

[1]Graduate Institute of Microbiology, College of Medicine, National Taiwan University, Taipei 100, Taiwan;

[2]Department of Neurology, National Taiwan University Hospital, Taipei 100, Taiwan;

[3]Department of Ophthalmology, Mackay Memorial Hospital, Taipei 104, Taiwan

[4] Graduate Institute of Medical Genomics and Proteomics, College of Medicine, National Taiwan University, Taipei 100, Taiwan

*Correspondence: Hsiang-Po Huang (hphuang691290@g.ntu.edu.tw) or Show-Li Chen (showlic@ntu.edu.tw)

These authors contributed equally: Hsiang-Po Huang, Show-Li Chen

Figure S1 Generation of NRIP-null C2C12 cells (KO19). (A) Schematic representation of the generation of NRIP-null cell lines via the CRISPR-Cas9 system. Trypsinized C2C12 cells (1×10^5 cells) were added with 2 μg plasmids (sgRNA-L-Cas9n-EGFP: sgRNA-R-Cas9n-puro = 3:1) and subjected to electroporation (1650 v/10 ms/3 pulses). Cells were seeded into 24-well plates containing antibiotic-free complete mediums, which were incubated overnight, and then selected with 3 μg/ml puromycin for two days. Then, the cells were diluted to one cell per well for single-cell cultures. After one-month, genomic DNA was harvested from half the cells per well for DNA sequencing. (B) The location of sgRNAs for NRIP gene editing. NRIP-sgRNA-F primer: GCC CGC ACC UGU UGU GGG AC; NRIP-sgRNA-R: CUU GGG CUG GAG GAC CCG UCC. The NRIP gene consists of nineteen exons with the translation starting site at exon 1. NRIP exon 1 was targeted by pairs of sgRNAs. (C) Sequences of genomic DNA from NRIP-null C2C12 cell lines and the locations of deletion sequences at the targeted NRIP from four NRIP-null cell lines. KO19 cells were chosen for the following assay.

Figure S2 The cell proliferation and transfection efficiency in KO19. (A) The cell proliferation in C2C12 and KO19 cells. The equal cell density (2×10^5 cells/dish) of C2C12 and KO19 cells were seeded in the 10-cm culture plate. The cell numbers were counted on the hemocytometer at 1, 2, and 3 days by trypan blue exclusion assay. C2C12, N=3 and KO19, N=4 for each day. Data are mean ± SD by student *t* test. (B) The transfection efficiency of Flag-NRIP in KO19 cells. The KO19 cells were transfected with Flag-NRIP and differentiated for eight days. The cells were stained with anti-flag (green) for Flag-NRIP expression and anti-MyHC (red) for myotube staining. The transfection efficiency was calculated as the Flag-NRIP$^+$ / MyHC$^+$ myotubes percentage to total MyHC$^+$ myotubes (N=3). The DAPI is the nuclear stain. Scale bar: 100 μm.

Figure S3 Silencing and depleting NRIP expression decreases the tip distribution of Tks5 in C2C12 myoblast. (A) The knockdown of NRIP expression reduces the tip distribution of Tks5. The C2C12 cells were transfected with pSuper-shNRIP plasmid for 24 h and shifted to differentiation medium for five days, then stained with Tks5 (Santa Cruz, red) and anti-NRIP (Abcam, green) antibodies and DAPI for nucleus (blue). Scale bar: 100 μm. Arrow: the enriched NRIP and Tks5 expression. Arrowhead: the dispersed Tks5 expression. Scale bar: 100 μm. (B) Average fluorescence intensity of Tks5 from the tip of NRIP-knockdown C2C12 cells and control C2C12 cells. Based on the immunofluorescence staining of Tks5, the Tks5 intensity was measured by circulating 480 μm^2 at the tip. (Control: 32.17, shNRIP: 15.75; N=3 for each group). Data are mean ± SD by student *t* test. *$P < 0.05$ (C) The Tks5 protein expression is comparable between NRIP-knockdown cells and wild-type cells (N=3). The GAPDH is a loading control. **(D) The total protein levels of Tks5 and cortactin are comparable between wild-type C2C12 cells and NRIP-knockout (KO19) cells transfected with either a control vector or Flag-NRIP. Endogenous NRIP and exogenous Flag-NRIP were detected using an anti-NRIP antibody, and GAPDH was used as a loading control.** (**E**) Depletion of NRIP expression in C2C12 (KO19) cells reduces the tip distribution of actin. The cells were differentiated for five days and then stained with anti-actin (red) and anti-NRIP (green) antibodies. Scale bar: 100 μm. Arrow: the enriched NRIP and actin localization. Arrowhead: the dispersed actin localization. Scale bar: 100 μm. Box: the tip of cells. (**F**) Average fluorescence intensity of actin from the tip of KO19 cells and C2C12 cells. The area of the tip is about 480 μm^2 in C2C12 cells and KO19 cells (C2C12: 45.77, KO19: 13.03; N=3 for each group). (**G**) The tip distribution of Tks5 in C2C12 cells and KO19 cells. The cells were differentiated for five days for IF staining. Arrow: the enriched NRIP and Tks5

localization. Arrowhead: the dispersed Tks5 localization. Scale bar: 100 μm. (**H**) Average fluorescence intensity of Tks5 from the tip of KO19 cells and control C2C12 cells. The area of the tip is about 480 μm^2 in C2C12 cells and KO19 cells (C2C12: 42.60, KO19: 12.50; N=3 for each group). Data are mean ± SD by student *t* test. $^{**}P < 0.01$

Figure S4 Analysis of NRIP-N mutant for actin interaction. (A) Schematic illustration of EGFP-tagged NRIP and NRIP deletion mutants. NRIP-FL (Full length), NRIP-N (N fragment containing the first five WD40 domains without IQ motif), NRIP-C (C fragment containing WD6/7 domains and one IQ motif). (B) NRIP-N mutant interacts with actin. 293T cells were transiently co-transfected with each NRIP truncated mutant and mCherry-actin plasmid for immunoprecipitation assay with anti-mCherry antibody. GAPDH as a loading control.

Figure S5 NRIP-actin binding correlates with invadosome formation in C2C12 using NRIP mutants' analysis. (A) NRIP localized at invadosome through actin interaction. C2C12 transfected with EFGP-NRIP mutants were stained with anti-EGFP (NRIP, green) and F-actin (red) antibodies at differentiation day 3 for IFA analysis. The deficient actin binding of NRIP mutant (C-ΔWD6/7ΔIQ) reduced the enriched localization at invadosome. All actin-binding NRIP mutants were significantly expressed at F-actin-enriched invadosome. DPAI (blue) for nuclear stain. (B) Quantification of NRIP mutants' localization at invadosome from panel E (NRIP-FL: 2.59, NRIPΔIQ: 2.19, NRIP-C: 2.23, C-ΔWD6/7: 2.42, C-ΔWD6/7ΔIQ: 0.91, NRIP-WD6/7: 3.23; N=6 for each group). The invadosome was measured the enrichment of NRIP with F-actin; hence the ratio was quantified by the intensity of NRIP in F-actin focus divided by the intensity outside the invadosome. Scale bars: 20 μm. Data are

mean ± SD. *$P < 0.05$; one-way ANOVA.

Figure S6 The mechanisms of NRIP involved in myotube formation through actin binding in C2C12 myotubes. (A) The comparable expression level of MyHC protein among C2C12 myotubes transfected with NRIP mutants. C2C12 myoblasts were transiently transfected with each EGFP-tagged NRIP mutant plasmid and differentiated for three days. The cell lysates were subjected to immunoblotting with anti-MyHC for MyHC protein expression, anti-EGFP for EGFP-tagged NRIP mutant expression, and anti-GAPDH for protein loading control. (B) The images of myotube formation. C2C12 were transfected with NRIP mutants including NRIP-FL, NRIPΔIQ, NRIP-C, C-ΔWD6/7, C-ΔWD6/7ΔIQ and NRIP-WD6/7, then subjected to immunofluorescence stain with anti-EGFP (NRIP, green), and anti-MyHC antibody (red). DAPI for nuclear stain (blue). The C-ΔWD6/7ΔIQ loss of actin-binding reduced myotube size. Scale bar: 100 μm. (C) Quantitation of NRIP mutants for myotube formation. The percentage of myotubes (≥ 5 nuclei in MyHC$^+$ cell) to total MyHC$^+$ cells was measured from each NRIP mutant (NRIP-FL: 65.18%, NRIPΔIQ: 62.53%, NRIP-C: 62.67%, C-ΔWD6/7: 56.00%, C-ΔWD6/7ΔIQ: 46.77%, NRIP-WD6/7: 64.51%) and shown on top of the box. The C-ΔWD6/7ΔIQ showed reduced MyHC$^+$ cells compared to NRIP-FL. The six data plots in each mutant were from six independent experiments (3 random fields in each experiment). Data are mean ± SD. **$P < 0.01$; ns, no significance; one-way ANOVA.

Figure S7 Analysis of the cellular localization of NRIP mutants in NRIP-knockout C2C12 (KO19) cells. (A) Immunofluorescence staining was performed to determine the localization of NRIP mutants in KO19 cells. The cells were transfected with EGFP-tagged NRIP mutants and subsequently subjected to immunofluorescence staining using an anti-EGFP (NRIP, green) antibody. DAPI was used for nuclear staining (blue). Scale bar: 5 μm. (B) Quantification of the

relative immunofluorescence intensity of NRIP mutants. Each NRIP mutant was normalized to NRIP-FL to determine the relative intensity ratio. The data for each mutant represent four independent experiments. Data are presented as mean ± SD. *$P < 0.05$; ns, not significant; one-way ANOVA.

Figure S8 Expression of endogenous NRIP and different AAV-NRIP mutant proteins in mouse muscles. The gastrocnemius muscles were dissected from wild-type (WT) mice and NRIP conditional knockout (cKO) mice infected with various AAV-NRIP mutants. Protein extracts were subjected to western blot analysis to assess the expression of endogenous NRIP, Flag-tagged NRIP-FL protein, and exogenous EGFP-tagged NRIP mutants. The expression of endogenous NRIP and Flag-tagged NRIP-FL was detected using an anti-NRIP antibody, while EGFP-tagged NRIP mutants were detected with an anti-EGFP antibody. GAPDH was used as a loading control.

Figure S9 The effect of AAV-NRIP mutants with actin-binding ability on the percentage of myotubes with central nuclei in NRIP cKO mice. The proportion of myotubes with central nuclei relative to the total myotubes was quantified in WT mice (N=4) and NRIP cKO mice treated with various AAV-NRIP mutants (N=6 for AAV-GFP and AAV-NRIP-FL; N=5 for AAV-NRIP-C, AAV-NRIP-C-ΔWD6/7, and AAV-NRIP-WD6/7). The significance of differences between the EGFP group and each of the other groups was assessed. Data are presented as mean ± SEM. *$P <$ 0.05, **$P < 0.01$, and ns, not significant; analyzed by one-way ANOVA.

Video S1 Time-lapse microscopy for NRIP and actin during invadosome structure formation (*Figure 4B*).

Fig. S1

A

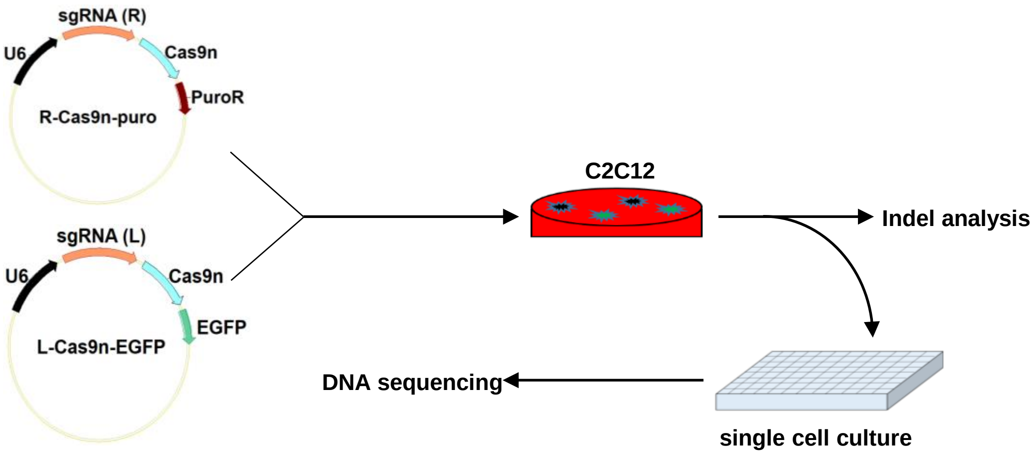

B

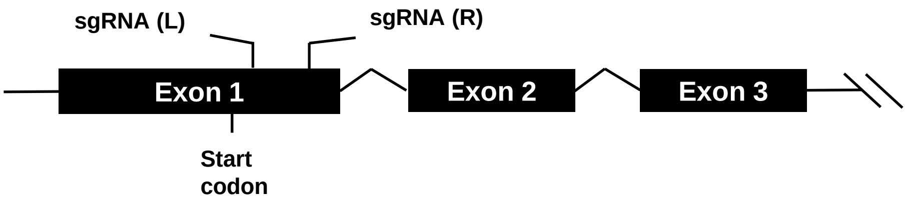

C

Sequences of genomic DNA from C2C12 following disruption of NRIP by CRISPR

Sample ID	Sequence across the deletion	Deletion (bp)	Cut by guide	Mutation
KO-3	GTTGTTCCT-----------CCGTCCCGG	31	guide (L) and (R)	Deletes D13-D23 and frame shift
KO-8	CCTGCCCG------------TGAGGAAAA	16	guide (L)	Deletes H9-V14 and frame shift
KO-12	CCTGCCCG-------------ACGTGA	13	guide (L)	Deletes H9-D13 and frame shift
KO-19	CCTGCCCGCAC------------AGGACC	37	guide (L) and (R)	Deletes L10-D23 and frame shift

Fig. S2

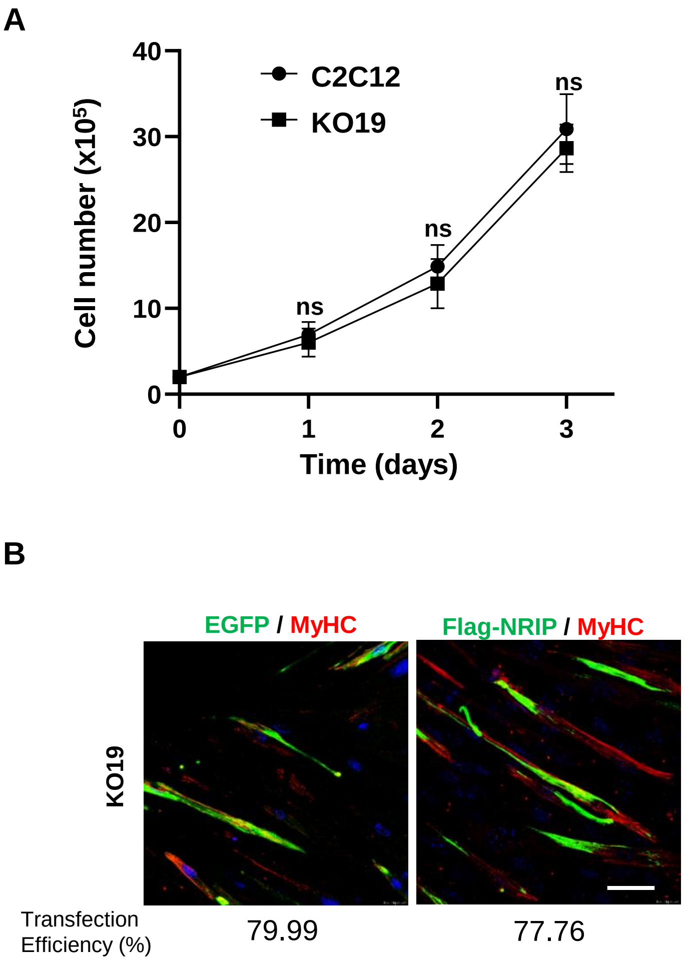
A
40
30
20
10
0
Cell number (x10^5)
C2C12
KO19
ns
ns
ns
0
1
2
3
Time (days)
B
EGFP / MyHC
Flag-NRIP / MyHC
KO19
Transfection Efficiency (%)
79.99
77.76

Fig. S3

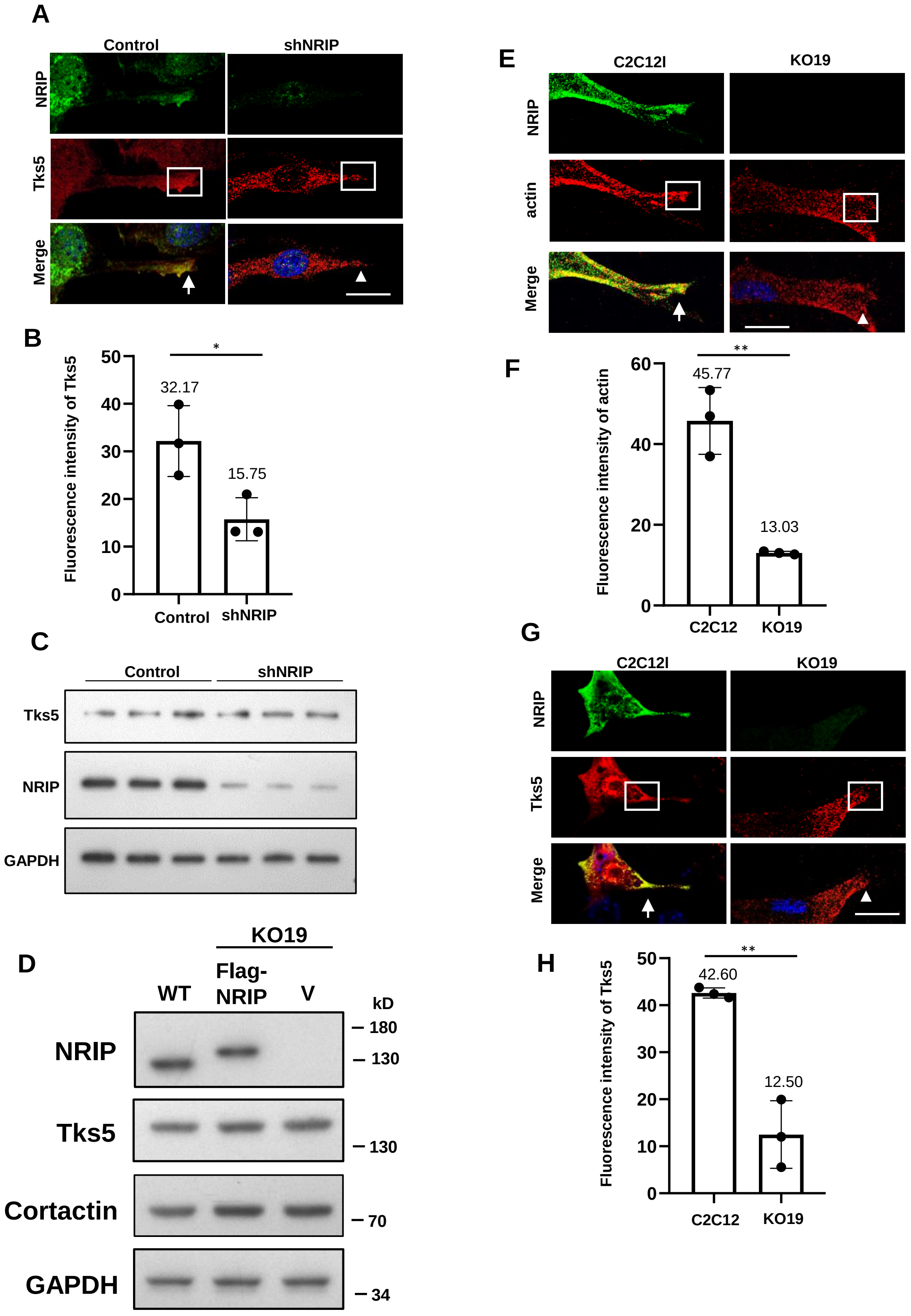

A
Control
shNRIP
NRIP
Tks5
Merge
B
Fluorescence intensity of Tks5
50
40
30
20
10
0
*
32.17
15.75
Control
shNRIP
C
Control
shNRIP
Tks5
NRIP
GAPDH
D
KO19
WT
Flag-NRIP
V
kD
NRIP
Tks5
Cortactin
GAPDH
180
130
130
70
34
E
C2C12I
KO19
NRIP
actin
Merge
F
Fluorescence intensity of actin
60
40
20
0
**
45.77
13.03
C2C12
KO19
G
C2C12I
KO19
NRIP
Tks5
Merge
H
Fluorescence intensity of Tks5
50
40
30
20
10
0
**
42.60
12.50
C2C12
KO19

Fig. S4

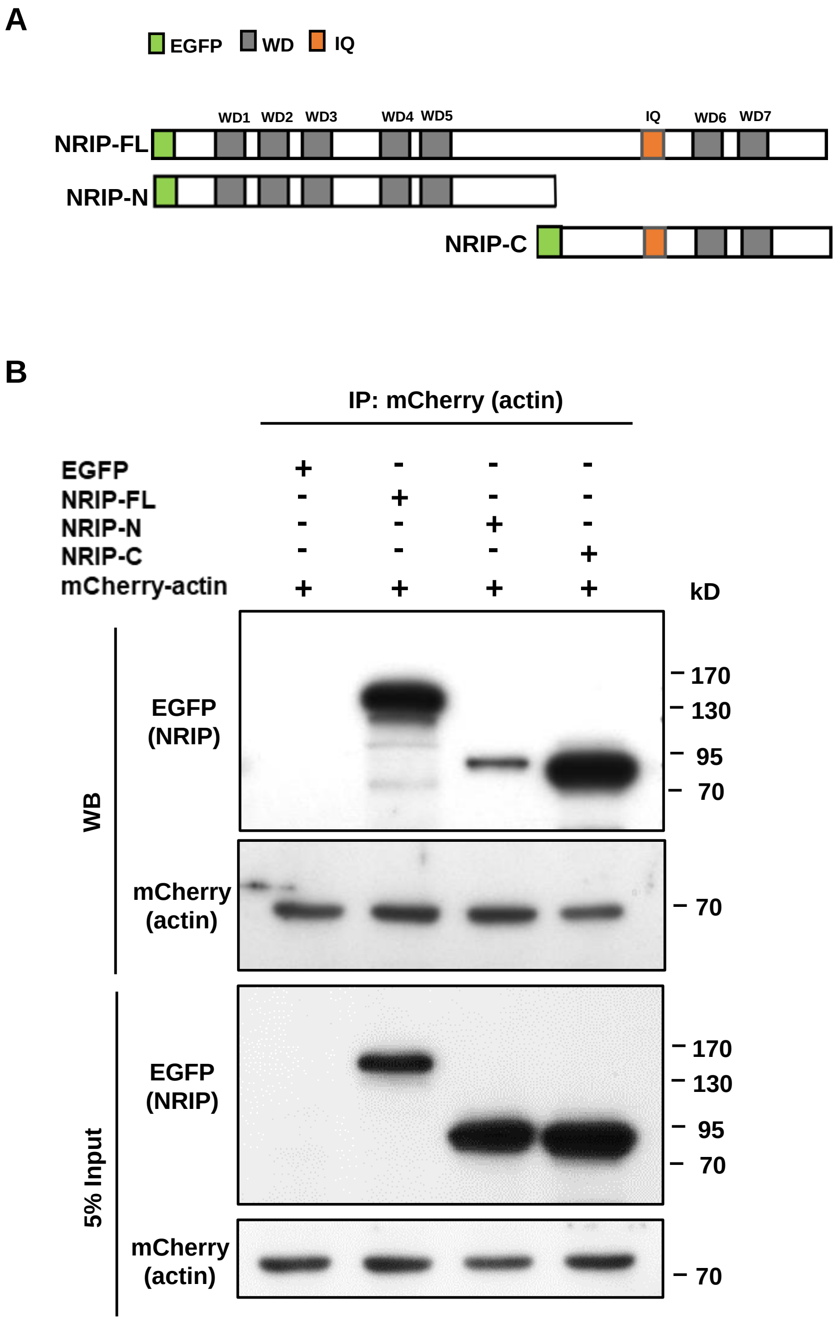
A
EGFP
WD
IQ
WD1
WD2
WD3
WD4
WD5
IQ
WD6
WD7
NRIP-FL
NRIP-N
NRIP-C
B
IP: mCherry (actin)
EGFP + - - -
NRIP-FL - + - -
NRIP-N - - + -
NRIP-C - - - +
mCherry-actin + + + +
kD
WB
EGFP (NRIP)
170
130
95
70
mCherry (actin)
70
5% Input
EGFP (NRIP)
170
130
95
70
mCherry (actin)
70

Fig. S5

A

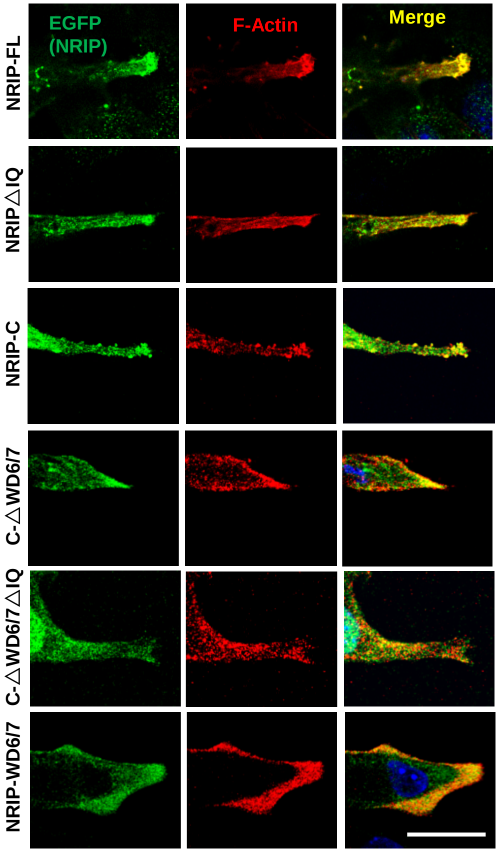
EGFP
(NRIP)
F-Actin
Merge
NRIP-FL
NRIP△IQ
NRIP-C
C-△WD6/7
C-△WD6/7△IQ
NRIP-WD6/7

B

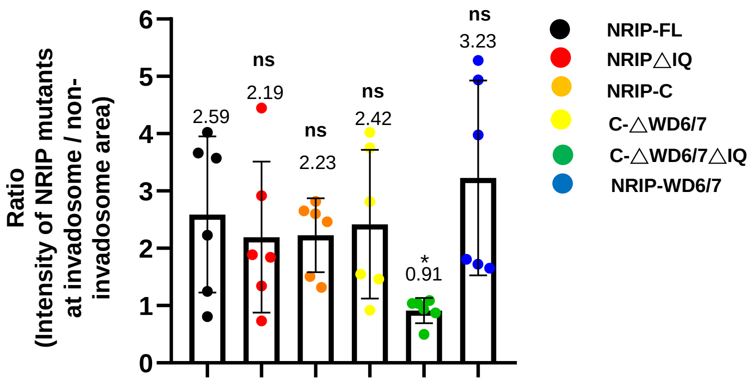
Ratio
(Intensity of NRIP mutants at invadosome / non-invadosome area)
0
1
2
3
4
5
6
2.59
ns
2.19
ns
2.23
ns
2.42
*
0.91
ns
3.23
NRIP-FL
NRIP△IQ
NRIP-C
C-△WD6/7
C-△WD6/7△IQ
NRIP-WD6/7

Fig. S6

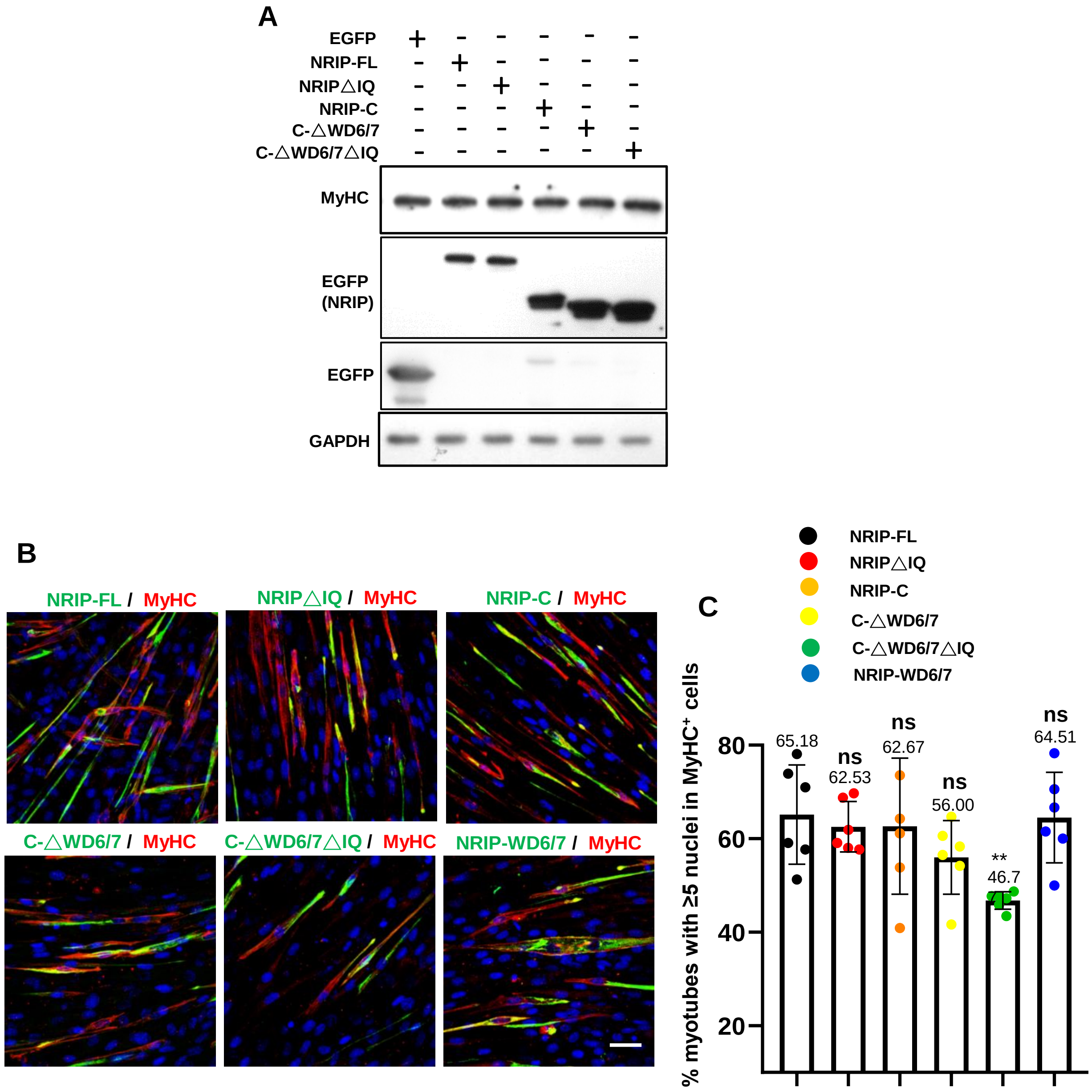
A
EGFP
NRIP-FL
NRIP△IQ
NRIP-C
C-△WD6/7
C-△WD6/7△IQ
MyHC
EGFP (NRIP)
EGFP
GAPDH
B
NRIP-FL / MyHC
NRIP△IQ / MyHC
NRIP-C / MyHC
C-△WD6/7 / MyHC
C-△WD6/7△IQ / MyHC
NRIP-WD6/7 / MyHC
C
NRIP-FL
NRIP△IQ
NRIP-C
C-△WD6/7
C-△WD6/7△IQ
NRIP-WD6/7
% myotubes with ≥5 nuclei in MyHC+ cells
80
60
40
20
65.18
ns
62.53
ns
62.67
ns
56.00
**
46.7
ns
64.51

Fig. S7

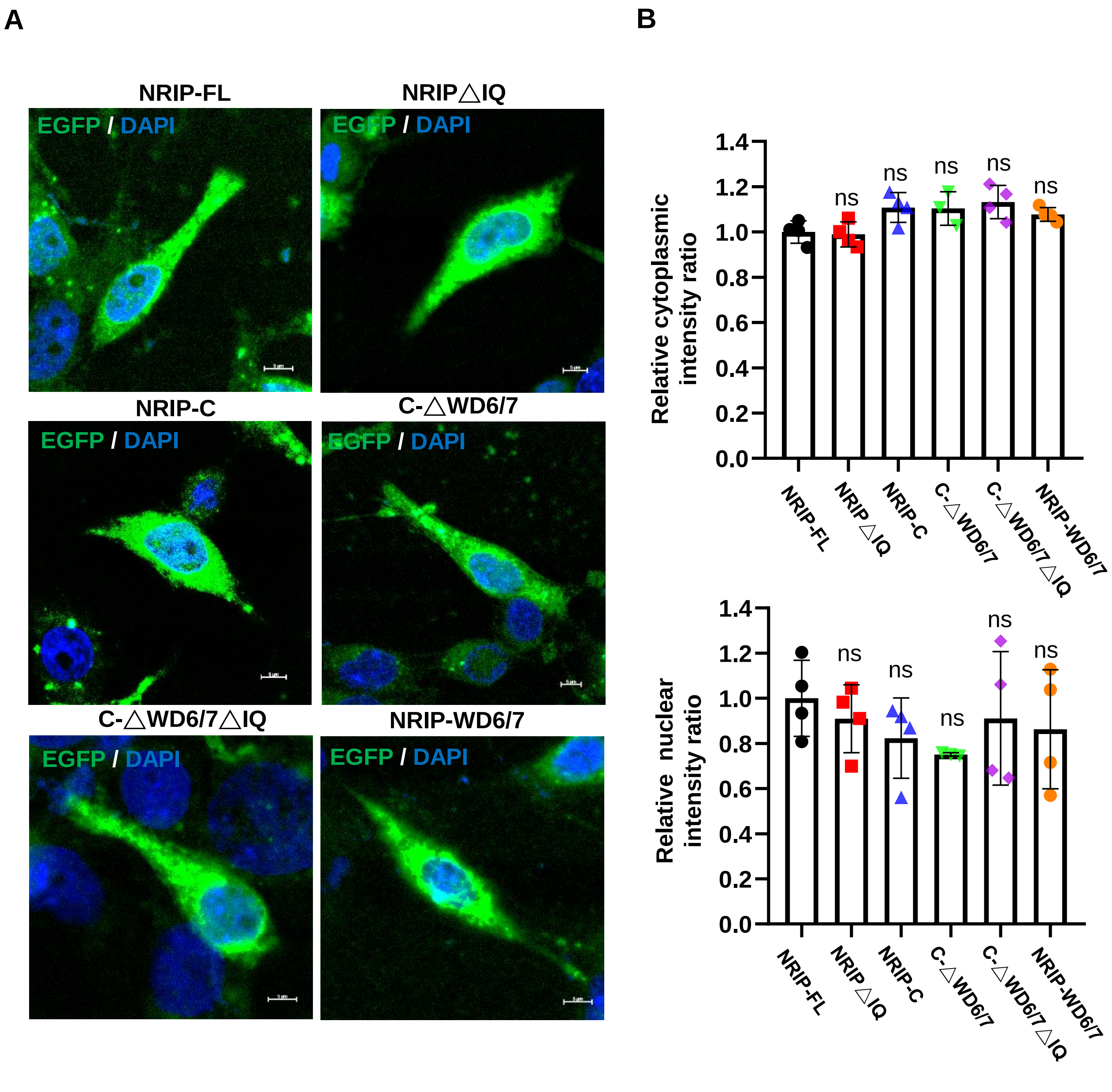
A
B
NRIP-FL
NRIP△IQ
NRIP-C
C-△WD6/7
C-△WD6/7△IQ
NRIP-WD6/7
EGFP / DAPI
Relative cytoplasmic intensity ratio
Relative nuclear intensity ratio
1.4
1.2
1.0
0.8
0.6
0.4
0.2
0.0
ns

Fig. S8

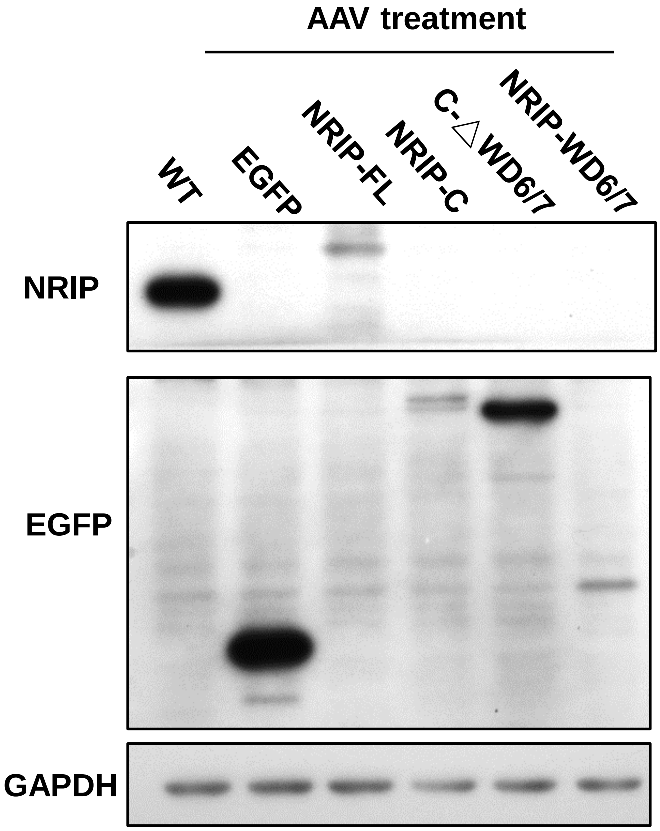

AAV treatment
WT
EGFP
NRIP-FL
NRIP-C
C-△WD6/7
NRIP-WD6/7
NRIP
EGFP
GAPDH

Fig. S9

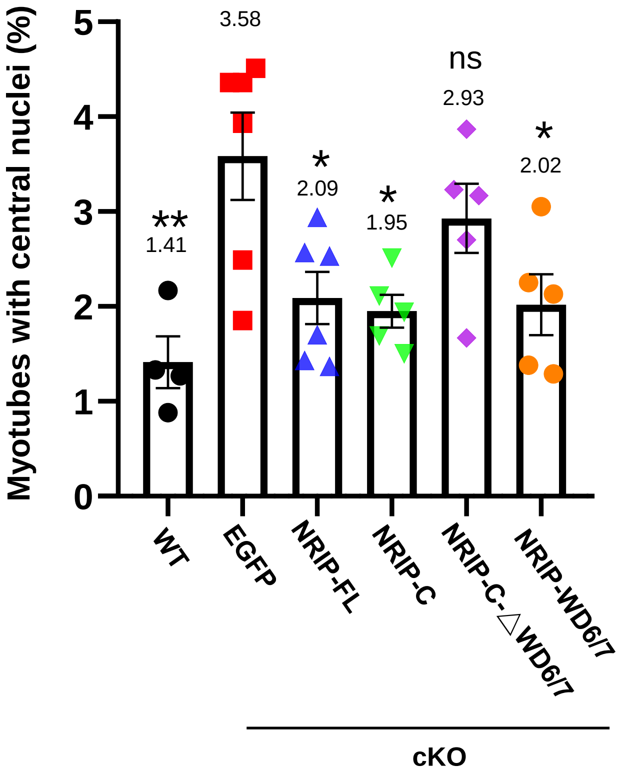

Myotubes with central nuclei (%)
5
4
3
2
1
0
3.58
ns
2.93
**
1.41
*
2.09
*
1.95
*
2.02
WT
EGFP
NRIP-FL
NRIP-C
NRIP-C-△WD6/7
NRIP-WD6/7
cKO
